# Lack of Offspring Nrf2 Does Not Exacerbate the Detrimental Metabolic Outcomes Caused by *In Utero* PCB126 Exposure

**DOI:** 10.3389/fendo.2021.777831

**Published:** 2021-12-16

**Authors:** Brittany B. Rice, Sara Y. Ngo Tenlep, Obadah Tolaymat, Attaas T. Alvi, Fallon R. Slone, Claire L. Crosby, Stevi S. Howard, Cecile L. Hermanns, Nishimwe P. Montessorie, Hollie I. Swanson, Kevin J. Pearson

**Affiliations:** Department of Pharmacology and Nutritional Sciences, University of Kentucky, Lexington, KY, United States

**Keywords:** developmental programming, diabetes, DOHAD, mice, nuclear factor erythroid-2-related factor 2 (Nrf2), obesity, pregnancy, polychlorinated biphenyals (PCBs)

## Abstract

Human environmental exposures to toxicants, such as polychlorinated biphenyls (PCBs), increase oxidative stress and disease susceptibility. Such exposures during pregnancy and/or nursing have been demonstrated to adversely affect offspring health outcomes. Nuclear factor erythroid-2-related factor 2 (Nrf2) regulates the antioxidant response and is involved in the detoxification of coplanar PCBs, like PCB126. The purpose of this study was to investigate glucose tolerance and body composition in PCB-exposed offspring expressing or lacking Nrf2. We hypothesized that offspring lacking Nrf2 expression would be more susceptible to the long-term health detriments associated with perinatal PCB exposure. During gestation, whole-body Nrf2 heterozygous (Het) and whole-body Nrf2 knockout (KO) mice were exposed to vehicle or PCB126. Shortly after birth, litters were cross-fostered to unexposed dams to prevent PCB exposure during nursing. Offspring were weaned, and their body weight, body composition, and glucose tolerance were recorded. At two months of age, PCB exposure resulted in a significant reduction in the average body weight of offspring born to Nrf2 Het dams (p < 0.001) that primarily arose from the decrease in average lean body mass in offspring (p < 0.001). There were no differences in average body weight of PCB-exposed offspring born to Nrf2 KO dams (p > 0.05), and this was because offspring of Nrf2 KO dams exposed to PCB126 during pregnancy experienced a significant elevation in fat mass (p = 0.002) that offset the significant reduction in average lean mass (p < 0.001). Regardless, the lack of Nrf2 expression in the offspring themselves did not enhance the differences observed. After an oral glucose challenge, PCB-exposed offspring exhibited significant impairments in glucose disposal and uptake (p < 0.05). Offspring born to Nrf2 Het dams exhibited these impairments at 30 min and 120 min, while offspring born to Nrf2 KO dams exhibited these impairments at zero, 15, 30, 60 and 120 min after the glucose challenge. Again, the interactions between offspring genotype and PCB exposure were not significant. These findings were largely consistent as the offspring reached four months of age and demonstrate that the lack of offspring Nrf2 expression does not worsen the metabolic derangements caused by *in utero* PCB exposure as we expected. Future directions will focus on understanding how the observed maternal Nrf2 genotypic differences can influence offspring metabolic responses to *in utero* PCB exposure.

## Introduction

Polychlorinated biphenyls (PCBs) are persistent halogenated organic pollutants that were synthesized for a host of commercial and industrial applications and products which include but are not limited to casting waxes, carbonless copy paper, paint, plastics, and inks (1). However, upon the realization of the health hazards posed from PCB exposure, production of the toxicants were halted but did not extinguish the presence of the detrimental pollutants as their lipophilic nature perpetuates their bioaccumulation and biomagnification (2). Thus, PCB exposure still occurs and health complications continue to persist (3). Environmental contamination from PCBs result from spills, leaks, and improper storage and disposal (4, 5). Once released, the environmental fates of PCBs are largely determined by their chlorination pattern (5) - as the chlorination pattern of the compound increases, so does its weight, viscosity, and lipophilicity (4). Routes of PCB exposure include inhalation, dermal contact, ingestion, and placental transfer (3, 6). Additionally, PCBs have been detected in umbilical cord and breastmilk and have been demonstrated to be transferred from mother to child during the perinatal period (6). It has been stated that the primary route of PCB exposure within the general population is primarily a result of the consumption of fatty foods such as dairy, animal products, and fish (7). Population studies demonstrate associations between coplanar PCBs and diabetes prevalence (8–10), incidence (11–13), and risk (14–16). In animal studies, such exposure has been shown to initiate and drive the development and progression of diabetes (17–23), and obesity (19, 22, 24–26). Existing evidence describing the contribution of early-life PCB exposure to diabetic-like phenotypes observed in mammalian offspring demonstrate sex-specific alterations in body composition (26) and metabolic parameters (27) as well as alterations in pro-inflammatory cytokines and hormones implicated in glucose regulation (28). However, such evidence fails to demonstrate the critical window of exposure during development that drives the long-term negative health outcomes or the genetic basis of observed phenotypes in mammalian species.

The harmful effects of coplanar PCBs, like 3, 3’, 4, 4’, 5-pentachlorobiphenyl (PCB126), are mostly elicited through its activation of the aryl hydrocarbon receptor (AhR) (29). This receptor is responsible for initiating the oxidation, reduction, and hydrolysis of xenobiotics in the detoxification pathway (30). The initial phase of reducing the toxicity of xenobiotics often results in the production of reactive oxygen species, which if not neutralized, can give rise to oxidative stress and damage (31). During the second phase of the detoxification pathway, xenobiotics and their respective metabolites are conjugated to increase their water-solubility for excretion (31). Genes involved in the second phase of detoxification are linked to the nuclear factor erythroid-2-related factor 2 (Nrf2) gene, which regulates antioxidant response by inducing the transcription of reactive oxygen species-detoxifying enzymes (32). Although the developmental role of Nrf2 in PCB-induced toxicity has been reported in zebrafish (33), these findings do not explain how Nrf2 allelic expression impacts diabetic-like phenotypes observed in mammalian offspring as a result of perinatal PCB exposure.

In the present study, we exposed Nrf2 heterozygous (Het) and Nrf2 knockout (KO) dams to PCB126 prior to and during pregnancy in an effort to delineate how both maternal and offspring Nrf2 genotype influences PCB-induced diabetic health outcomes in offspring. Dams were mated with sires of opposing Het and KO genotypes. Offspring body weight and body composition as well as glucose tolerance were monitored. Data collected from the offspring were analyzed from the perspective of offspring and maternal Nrf2 genotype and treatment, not paternal genotype. We hypothesized that PCB-induced detriments in offspring would be exacerbated by the lack of Nrf2 expression in offspring. We found that gestational PCB126 exposure precipitates adverse offspring metabolic and phenotypic responses but that offspring responses were modulated by maternal Nrf2 genotype, not offspring genotype.

## Materials and Methods

### Animals Care, Husbandry, Cross-Fostering, and Weaning

**Figure 1:**
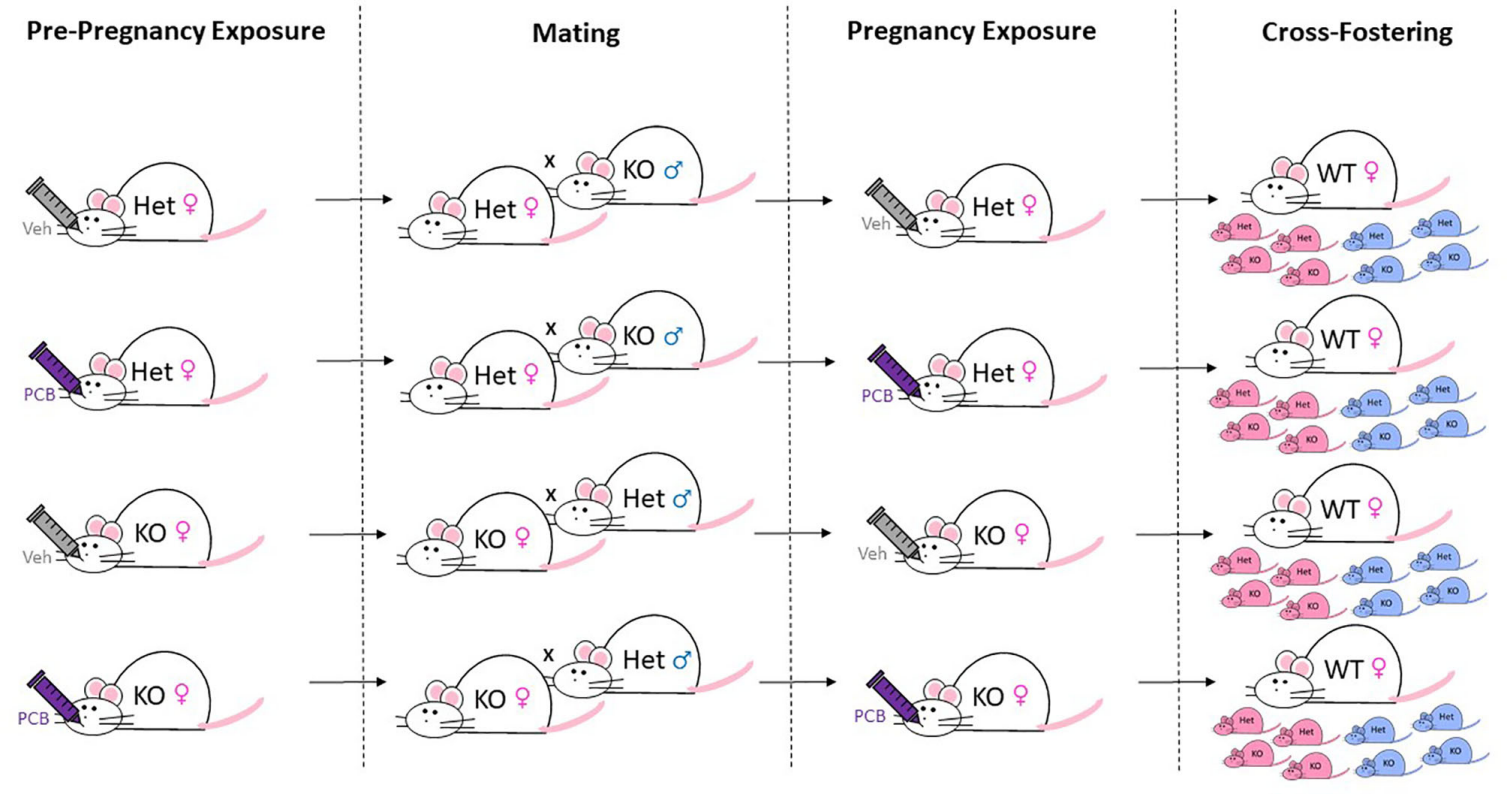
Breeding, Exposure, and Cross-Fostering Scheme. Whole-body Nrf2 heterozygous (+/-) and Nrf2 knockout (-/-) female ICR mice were exposed to 1 µmol/kg of PCB126 or safflower (vehicle) three days prior to mating and on gestation day ~10. For breeding, female mice were housed with male mice of opposing Nrf2 genotype for a week. Shortly after birth, litters containing more than eight pups underwent culling to ensure postnatal food availability. Remaining offspring were then cross-fostered to unexposed Nrf2 wild-type dams to prevent PCB exposure during nursing. The figure denotes 50% Het and 50% KO (as well as male and female) offspring as an example for each litter, but actual litters did not always have this breakdown of sex/genotypes.

All experimental procedures were approved by the University of Kentucky Institutional Animal Care and Use Committee. Throughout the study, housing conditions included a 14:10 light/dark cycle, temperature ranging from 20 – 22.2˚ Celsius, and humidity ranging from 30% to 70%. All mice were given *ad libitum* access to chow (24% kcal from protein, 6.2% kcal from fat, and 44.2% kcal from carbohydrate; #2918; Teklad Diets; Envigo, Madison, WI) and water. Additionally, nesting material (Nestlet; Ancare Corporation; Bellmore, NY) was placed in animal caging throughout the duration of the study. Animals were singly housed during the acclimation period. Whole-body Nrf2 Het and KO dams and sires used in this experiment were generated from repeated in-house breeding of Nrf2 Het mice on an ICR background generously gifted by Dr. Viviana Perez upon permission from Dr. Masayuki Yamamoto who originally developed the line as previously described (34). The use of the Het mice was based on rationale from our preliminary work where whole-body Het and wild-type (WT) non-pregnant females exposed to 1 µmole of PCB126 per kg body weight or vehicle did not differ in glucose response upon an intraperitoneal glucose challenge (see **Supplementary Figure 1**). At 18 weeks old, whole-body Nrf2 Het (n = 28) and KO (n = 52) female ICR mice were mated with whole-body Nrf2 Het or KO male ICR mice of opposing genotype. Simultaneously, foster dams were created by breeding WT male and female (n = 80) ICR mice (Envigo-Harlan; Indianapolis, IN). Breeding schemes consisted of mating pairs and/or trios. All dams were exposed to male bedding 48 hours prior to mating to stimulate estrus. Sires were allowed to mate with dams for one week. On postnatal day 0, all offspring of WT dams were culled, while offspring of Het and KO dams were cross-fostered to WT dams. See **Figure 1** for an illustration of animal husbandry and cross-fostering. Larger litters were reduced to eight pups and pup sex ratios per litter were kept equal when possible. Offspring weaning occurred at three weeks of age. After weaning, mice were housed two to five animals per cage per sex and, in most cases, per litter.

### Chemicals and Exposure

Using weight matching, Het and KO dams were assigned to treatment groups. Three days prior to mating and on gestation day ~10 (this is an estimate as breeding pairs were allowed to mate for one week), Het and KO dams were orally administered vehicle (tocopherol-stripped safflower oil, Dyets; Bethlehem, PA) or 1 μmole of PCB126 per kg body weight (AccuStandard Inc.; New Haven, CT) dissolved in vehicle according to their groupings (Het Veh, n = 14; Het PCB, n = 14; KO Veh, n = 26; KO PCB, n = 26). The rationale for this dosing paradigm is based upon our previous work (26) where we conducted a dosing study to establish a mouse model in order to study the mechanism(s) of and interventions to protect against perinatal exposures to PCB126 in future experiments. Further, it is important to note that human exposure to toxicants, like PCB126, occur at low levels over the life course. Thus, our mouse model attempts to recapitulate their effects in a relatively short exposure study.

### Offspring Genotyping

Ear punches were collected from pups, and DNA was isolated, washed, and purified according to the manufacturer’s instructions (Maxwell^®^ 16 Mouse Tail DNA Purification Kit; Promega; Madison, WI). Polymerase chain reaction was performed (C1000™ Thermal Cycler; Bio-Rad; Hercules, CA) using 1 μl of each eluted DNA sample, 10 μl of GoTaq^®^ Master Mix (Promega; Madison, WI), deionized water, and the following three primers (Integrated DNA Technologies, Inc.; Coralville, IA): (1) Nrf2-5, 5’ TGG ACG GGA CTA TTG AAG GCT G 3’; (2) Nrf-LacZ, 5’ GCG GAT TGA CCG TAA TGG GAT AGG 3’; (3) Nrf2 AS, 5’ GCC GCC TTT TCA GTA GAT GGA GG 3’. The cycling conditions were as follows: initial denaturation at 94°F for 5 minutes, followed by 35 cycles at 94°F for 30 seconds, 61°F for 30 seconds, and 72°F for 30 seconds. PCR products as well as 100 bp DNA ladder (Invitrogen; Waltham, MA) were then loaded into a 1.2% gel consisting of UltraPure™ Agarose (Invitrogen), SYBR™ Safe DNA Gel Stain (ThermoFisher Scientific; Waltham, MA), and TBE Buffer 10x (Promega; Madison, WI). Subsequently, gel electrophoresis was performed at 100 V for 30 minutes (Powerpac HC; Bio-Rad; Hercules, CA). Nrf2 KO mice-derived PCR products showed one band of 400 base pairs, while Nrf2 Het mice-derived PCR products showed two bands at 400 and 734 base pairs.

### Offspring Phenotypic and Metabolic Assessments

Offspring body weight was recorded weekly. Total fat tissue, lean tissue, and water were measured in live, conscious offspring by nuclear magnetic resonance (EchoMRI; Echo Medical Systems, Houston, TX) at two and four months of age. Animals that underwent body composition analyses were initially selected at random and used for both assessments when possible. Specifically, two Het and two KO offspring per sex of each litter were selected and the average of the two offspring are presented when possible. In the infrequent event that an animal was excluded from the assessment for treatment-independent health concerns, a littermate of that animal of the same genotype was selected to take the place of the excluded animal.

For oral glucose tolerance testing, animals were fasted for three hours then administered 2 g of dextrose solution (VetOne, Nova-Tech, Inc.; Grand Island, NE) per kg body weight at both two and four months of age. A subset of offspring, one Het and one KO offspring per sex per each dam of each experimental grouping (Het Veh, Het PCB, KO Veh, and KO PCB) were selected when possible. Glucose levels were measured from blood collected from the tail vein at fasting and at 15, 30, 60, and 120 minutes after the glucose challenge. AUC for blood glucose measurements were calculated in SigmaPlot 14.0 (Inpixon; Palo Alto, CA) using the ‘Area Below Curves’ function.

### Statistics

All analyses were completed using SigmaPlot 14.0. Chi-square tests were performed to determine if associations between dam experimental groupings and the number of litters born or weaned existed. Contingency tables for Chi-square analyses were modified slightly from **Table 1** and are described below. In testing associations between dam groupings and number of litters, categorical variables of no litter and litters were assigned. Values for no litters were obtained by subtracting values for litters born from females bred respective to each dam experimental grouping in **Table 1**. Initial testing of associations between dam groupings and litters weaned used values from litters born and values from litters weaned shown in **Table 1**. However, due to low incidence of litter death, Chi-square analysis was not completed because at least one of the expected values in our contingency table was less than one and over 20% of the values were less than five. Thus, we determined associations between dam experimental groupings and litters weaned by using females bred and litters weaned as categorical variables. The effects of sex, genotype, and/or treatment were individually and collectively evaluated. Litter size was assessed using two-factor ANOVA. Offspring body weight, body composition, glucose tolerance, and AUC were assessed using three-factor ANOVA. When interactions were detected, Fisher’s Least Significant Difference *post-hoc* testing was employed. Statistical significance for all comparisons was set at 0.05. The distribution of the data was measured using Shapiro-Wilk normality test, while Brown-Forsythe equal variance test was used to measure the spread of the data. Data that did not present normal distributions and/or spreads were transformed to values of natural log. When appropriate, transformations were used to improve assessment of normality and equal variance.

**Table 1:** Pregnancy and rearing information.

Group	Females Bred^a^	Litters Born^a^	Litters Weaned^a^	Mean Litter Size^b^ (SEM)
Het Veh	14	8	8	11 (0.78)
Het PCB	14	10	10	11 (0.93)
KO Veh	26	13	12	12 (0.63)
KO PCB	26	16	13	10 (0.64)

a No associations detected between Females Bred and Litters Born or Litters Weaned using Chi Square Test of Independence.

b Using two-factor ANOVA, no differences in or interactions between genotype and treatment detected in Mean Litter Size.

## Results

### Maternal Pregnancy and Rearing

The number of litters born and weaned from each dam experimental grouping was recorded on postnatal day 0 and 21, respectively. Because p-values for litters born (p = 0.604) and litters weaned (p = 0.462) obtained were greater than the significance level of 0.05, no associations between dam genotype and treatment and the number of litters born and weaned were detected (**Table 1**). Neither dam genotype nor treatment influenced litter size (**Table 1**; p = 0.842 and 0.255, respectively). Further, no interactions between genotype and treatment were observed in regards to litter size (**Table 1**; p = 0.353).

### Offspring Body Weight and Body Composition

**Figure 2:**
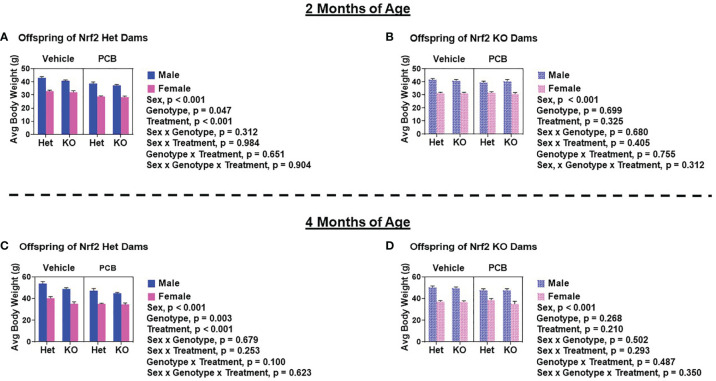
Adult offspring born to Nrf2 heterozygous dams exposed to PCB126 during pregnancy are more sensitive to the adverse effects of PCB126 on body weight. Offspring body weight was recorded weekly. Displayed are the body weights of offspring at two and four months of age that were born to Nrf2 heterozygous **(A, C)** and knockout **(B, D)** dams exposed to PCB126 or vehicle during pregnancy. Average offspring body weight values are representative of the mean of litter means (n = 8 – 13 per group) ± SEM. Data were analyzed using three-factor ANOVA. Significance was set at α = 0.05.

**Figure 3:**
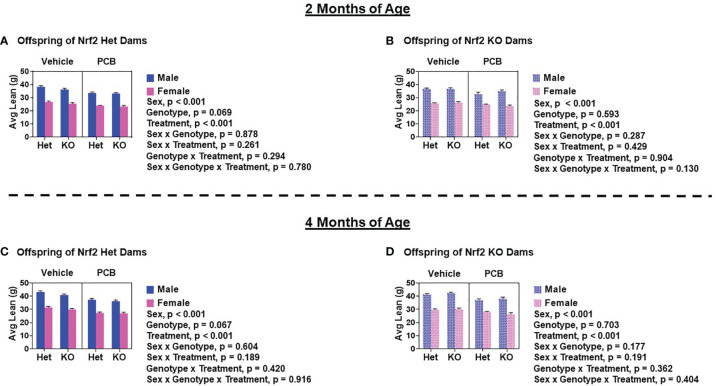
PCB126 exposure during gestation adversely influences lean mass profiles in adult offspring. Displayed average lean mass measurements of offspring of Nrf2 heterozygous **(A, C)** and knockout **(B, D)** dams at two and four months of age were taken using EchoMRI. Values are representative of the mean of litter means (n = 8 – 13 per group) ± SEM. Three-factor ANOVA was used to analyze data. Significance was set at α = 0.05.

**Figure 4:**
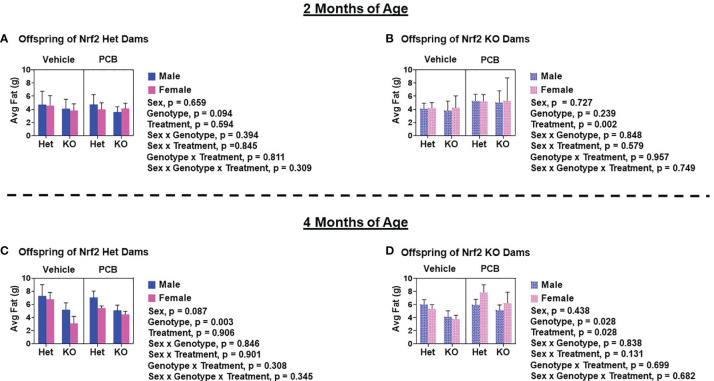
Adult offspring born to Nrf2 knockout dams exhibit significant elevations in fat deposition as a result of gestational PCB126 exposure. Shown are the fat mass measurements of offspring born to Nrf2 heterozygous **(A, C)** or knockout **(B, D)** dams at two and four months of age. Data were obtained using EchoMRI and the values shown are indicative of the mean of litter means (n = 8 – 13 per group) ± SEM. Offspring fat mass data were analyzed using three-factor ANOVA. Significance was set at α = 0.05.

At two and four months of age, male offspring weighed significantly more than female offspring irrespective of maternal Nrf2 genotype (**Figures 2A**–**D**; p < 0.001). A main effect of offspring genotype in average body weight was only observed in offspring born to Het dams (**Figures 2A**, p = 0.047; 2C, p = 0.003), where KO offspring weighed significantly less than Het offspring (**Figures 2A, C**). Additionally, a main effect of PCB treatment in average body weight was only observed in offspring born to Het dams (p < 0.001), where offspring exposed to PCB126 during gestation weighed significantly less than vehicle exposed offspring (**Figures 2A, C**). No interactions between groups were detected (**Figures 2A**–**D**; p > 0.05); suggesting that outcomes in offspring exposed to PCBs did not differ depending on their genotype. Male offspring had significantly elevated average lean mass when compared to female offspring (**Figures 3A**–**D**; p < 0.001), while no sex differences were observed in offspring fat mass (**Figures 4A**–**D**; p > 0.05). At two months of age, offspring genotype did not influence body composition profiles (fat and lean mass, p > 0.05). However, at four months of age, offspring genotype did alter offspring fat deposition (**Figures 4C, D**; p < 0.05), where Het offspring had significantly elevated fat mass profiles when compared to KO offspring. At two and four months of age, exposure to PCB126 during gestation caused a significant decrease in the lean mass of offspring born to Het dams (**Figures 3A, C**; p < 0.001) and KO dams (**Figures 3B, D**; p < 0.001) while producing an increase in fat mass only in offspring born to KO dams (**Figures 4B, D**; p < 0.05).

### Offspring Glucose Tolerance

**Figure 5:**
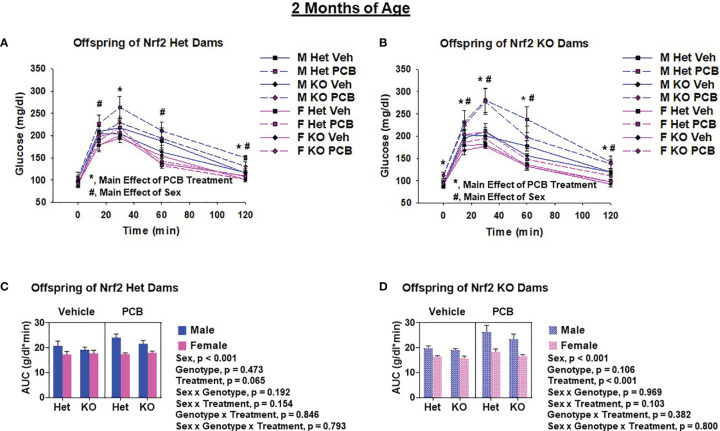
Two-month-old offspring exposed to PCB126 during gestation exhibit impairments in glucose tolerance. At two months of age, glucose was measured in Nrf2 heterozygous and knockout offspring exposed to PCB126 or vehicle during gestation in response to an oral glucose challenge **(A, B)**. Glucose measurements were then used to determine area under the curve (AUC; **C, D**). Offspring glucose and AUC values are representative of the mean of litter means (n = 8 per group) ± SEM. Three-factor ANOVA at each time point was used to detect differences between groups. Significance was set at α = 0.05. For simplicity, only significant main effects of PCB treatment (*) and sex (#) are shown at each time point.

**Figure 6:**
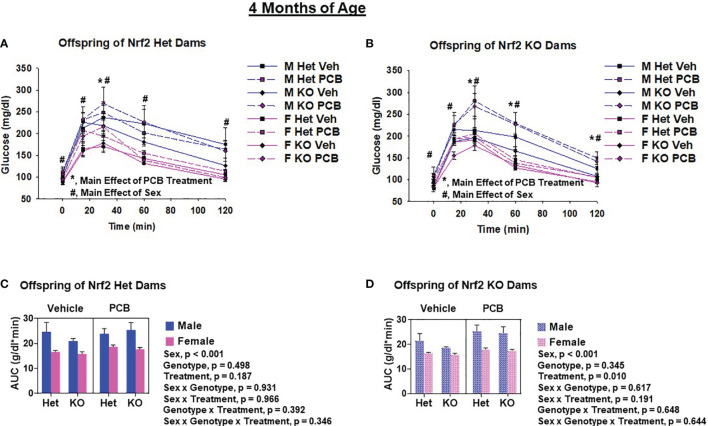
PCB126 gestational exposure perturbs offspring glucose homeostasis profiles at four months of age. At four months of age, glucose was measured in Nrf2 heterozygous and knockout offspring exposed to PCB126 or vehicle during gestation in response to an oral glucose challenge **(A, B)**. Glucose measurements were then used to determine area under the curve (AUC; **C, D**). Values reported represent the mean of litter means (n = 8 per group) ± SEM. Differences between groups were detected by three-factor ANOVA. Significance was set at α = 0.05. For simplicity, only significant main effects of PCB treatment (*) and sex (#) are shown at each time point.

Glucose tolerance tests were performed to assess offspring glucose homeostasis. At two months of age, adult offspring of Het dams exposed to PCB126 during pregnancy had significantly elevated blood glucose levels at 30 min and 120 min (**Figure 5A**, p < 0.05 in both comparisons) when compared to offspring from vehicle exposed dams, whereas offspring of KO dams exposed to PCB126 during pregnancy presented higher glucose levels at each time point during testing when compared to offspring from vehicle exposed dams (**Figure 5B**, p < 0.05 in all cases). At four months of age, significant elevations in the blood glucose levels of adult offspring of Het dams exposed to PCB126 during pregnancy persisted only at 30 min (**Figure 6A**, p < 0.05) when compared to offspring from vehicle-exposed dams, while significant elevations in the blood glucose levels of offspring of KO dams exposed to PCB126 pregnancy remained at 30, 60, and 120 min (**Figure 6B**, p < 0.05) when compared to offspring born to vehicle-exposed dams. Analyses revealed significant sex differences in blood levels of glucose following the glucose challenge. Specifically, male offspring of Het dams exhibited significantly impaired glucose tolerance when compared to female offspring of Het dams at 15 min (p < 0.05), 60 min (p < 0.001), and 120 min (p < 0.001) (**Figure 5A**). Male offspring of KO dams exhibited significantly impaired glucose tolerance when compared to female offspring of KO dams at each time point beyond fasting (p < 0.05) (**Figure 5B**). Interactions between sex and treatment were only detected at two months of age. In adult offspring born to Het dams, at 120 min, males exposed to PCB126 during gestation had significantly higher glucose levels than females exposed to PCB126 during gestation or males exposed to vehicle (p < 0.01) (**Figure 5A**). The fasting blood glucose levels of adult male offspring born to KO dams exposed to PCB126 during pregnancy was more pronounced than adult female offspring exposed to PCB126 during gestation or males exposed to vehicle (p < 0.05) (**Figure 5B**). Of note, there were no significant interactions between PCB and offspring genotype.

The AUC was used to summarize the glucose disposal over the 120 minute time course of the glucose tolerance test. While PCB treatment caused a trend toward impaired glucose disposal in two-month-old offspring born to Het dams (**Figure 5C**, p = 0.065), the PCB treatment caused a significant difference in glucose disposal in offspring born to KO dams at both two and four months of age (**Figures 5D**, **6D**; p < 0.05). Of note, there was no significant interaction between PCB treatment and offspring genotype in either comparison (**Figures 5C, D**, **6C, D**). There were sex differences where male offspring present significantly elevated AUCs compared to female offspring (**Figures 5C, D**, **6C, D**; p < 0.001), but there were no significant differences in offspring genotype (**Figures 4C, D**; p > 0.05).

## Discussion

Nrf2 has been identified as an integral regulator of redox homeostasis, and therefore its associated signaling pathway has been widely explored as a therapeutic target towards the prevention of a host of diseases (35, 36), including diabetes (37). Emerging data implicates the involvement of PCB exposure in the development and progression of diabetes (14–16). Unfortunately, literature insufficiently details the role of Nrf2 expression in PCB-induced diabetes. The current study focused on delineating the effects of Nrf2 allelic expression on offspring phenotypic and metabolic responses to gestational PCB126 exposure. Unpublished data from our laboratory demonstrates that PCB treatment during the nursing period did not significantly affect offspring body weight, body composition, or long-term glucose intolerance. Here, using a cross-fostering strategy, we provide additional evidence that the *in utero* period is the critical window that precipitates long-term negative developmental programming of altered body composition and impaired glucose tolerance in offspring. To our surprise, we found that maternal rather than offspring Nrf2 genotype impacted long-term PCB-induced developmental programming in offspring where offspring born to KO dams seemed to be more sensitive to PCB126.

Although little is known about PCB-induced diabetes and obesity resulting from developmental exposure, we expected offspring body weight and composition as well as glucose tolerance would be adversely affected by gestational PCB exposure. Our anticipation was largely based on studies in which direct PCB exposure occurred. Animals directly exposed to PCBs exhibit pronounced elevations in body weight as a result of exposure (22, 38–40). Moreover, limited data detailing the effects of direct PCB exposure on body composition demonstrate conflicting results and utilize diet as a means to understand the influence of PCB exposure on fat and lean mass (20, 39). Nonetheless, PCBs have been demonstrated to alter adipocyte development (21, 24, 25, 41) and death (25), and as a result have been deemed as obesogens. Further, direct PCB exposure has been shown to induce impairments in glucose (17, 18, 21, 23, 39, 40, 42) and insulin (21, 22, 39) tolerance. Previously, in seven-week-old offspring exposed to PCB126 during pregnancy and nursing, we observed that toxicant exposure did not influence offspring body weight but did alter offspring body composition in a sex- and dose-dependent manner (26). Furthermore, our unpublished data demonstrates that exposure to PCB126 exclusively during nursing does not affect offspring body weight or body composition but does affect offspring early-life glucose tolerance in a sex-dependent manner. Mennigen et al. (43) reported that gestational exposure to Aroclor 1221, a commercial PCB mixture, did not influence offspring pre-weaning and adolescent body weights. Dioxin exposure during pregnancy was reported to not affect offspring body weight or offspring later-life glucose and insulin tolerance (44). Perinatal PCB153 exposure has been shown to alter offspring glucose homeostasis in a sex-dependent manner, where male offspring experience elevated blood glucose levels and female offspring experience elevated glucagon levels (27). Although findings regarding the perinatal influence of PCB exposure on offspring body weight differ and evidence scantly describes the effects of such exposure on offspring body composition and glucose homeostasis, our current findings support and extend our earlier work (26) and reiterate the importance of timing of exposure in regards to offspring diabetes and obesity risk. Our current results demonstrate that gestational PCB126 exposure alters offspring body weight and composition as well as impairs glucose tolerance.

Interpretations of our aforementioned findings slightly vary when taking Nrf2 genotype into consideration. Nrf2 acts as an endogenous defense whose downstream mechanisms combat and reduce excess oxidants produced from xenobiotic insults, such as PCB exposure. Specifically, Nrf2 regulates the chemical induction of a number of detoxification pathway Phase II enzymes, which include but are not limited to glutathione peroxidase, heme oxygenase 1, and NAD(P)H dehydrogenase quinone 1 (NQO1) (45, 46). While we did not measure the gene expression of Nrf2 target genes in PCB-exposed offspring of the current study, we observed pronounced elevations in the hepatic mRNA expression of NQO1 in six-week-old WT and whole-body Nrf2 Het mice directly exposed to PCB126 in a separate study. Further, NQO1 levels were significantly reduced in both male and female KO mice compared to WT mice (**Supplementary Figure 2**). Because of the aforementioned findings in addition to the known importance of Phase II response in the detoxification of PCBs, in the current study, we hypothesized that offspring lacking Nrf2 expression would be more susceptible to the long-term health detriments associated with *in utero* PCB exposure. Rather, we observed that there were no significant interactions between offspring genotype and perinatal PCB exposure. Instead, we found that offspring of KO dams exposed to PCB126 during gestation are more sensitive to the long-term negative consequences of PCB exposure than offspring of Het dams that were exposed to PCB126 during gestation. PCB-exposed offspring born to KO dams had increased fat mass and significantly impaired glucose disposal whereas PCB-exposed offspring born to Het dams did not (**Figures 4**–**6**). The developmental role of Nrf2 in PCB-induced toxicity has been previously described in zebrafish (33); however, this does not extend to rodent models. To our knowledge, the present study is the first to report the developmental role of Nrf2 in PCB-induced toxicity in rodents. Previously, mice have been utilized to delineate the developmental role of Nrf2 in neural function and/or behavior in response to pharmaceutical (47) and recreational drugs (48), antioxidants (49), as well as heavy metals (49). Diet studies have been used to better understand the role of Nrf2 expression in obesity and diabetes. Studies investigating the effects of Nrf2 deficiency on body weight and composition report divergent results. Nrf2 deficiency in animals on standard or chow fed diets have been shown to have comparable body weights (50–53) and body fat compositions (50) to WT and/or Nrf2 mice with enhanced activity, as well as lower early-life body weights when compared to their WT littermates (54). Differences in body weights reported by Pi et al. (54) are likely due to the decreased adipose tissue mass possessed by Nrf2 KO animals as they also report the ability of Nrf2 deficiency to impair adipogenesis and reduce susceptibility to diet-induced obesity. Similarly, conflicting results have been reported about the influence of Nrf2 deficiency in the presence of a high fat diet (HFD). It has been stated that deficient animals fed a HFD have lower body weight and less weight gain than WT mice (51), as well as no differences in body weight and body fat composition (50). In diabetic mice, Nrf2 deficiency worsens hyperglycemia (55). However, in the presence of a HFD, Nrf2 deficiency improves glucose tolerance when compared to WT and/or Nrf2 mice with enhanced activity (51, 53). Conversely, it has been reported that a HFD impairs glucose tolerance in both WT and KO animals, where the deficiency worsens the observed impairment (52). The findings from the aforementioned studies demonstrate the dual roles of Nrf2 in obesity and insulin resistance. While the present study did not investigate the influence of Nrf2 expression on diabetes and obesity from a dietary standpoint, PCB126 exposure could be considered a ‘second-hit’ similar to HFD feeding. Our findings demonstrate the ability of Nrf2 genotype, maternal genotype in this case, to modulate PCB-induced diabetes and obesity.

We were surprised to find that the influence of maternal Nrf2 genotype on offspring metabolic and phenotypic responses to gestational PCB exposure supersedes that of the offspring Nrf2 genotype. To better understand this finding, we conducted a literature search on the effects of maternal genotype on offspring response respective and irrespective to environmental contaminant exposure. Obtained search results were not relevant and further demonstrate the need to investigate the influence of the maternal gene-toxicant exposure interactions on offspring response. Thus, we propose that our future studies examining this phenomenon will measure PCB126 parent compound levels in dams throughout pregnancy, in fetuses, and in offspring throughout the lifecycle with the hopes of understanding more about placental PCB transfer respective to Nrf2 genotypes and its short- and long-term effects on offspring response. Our preliminary findings in non-pregnant females with varying Nrf2 genotypes (WT, Het, and KO) showed no differences in sera and liver PCB126 parent compound measurements (data not shown). These data suggest that genotype does not influence PCB126 levels; however, this does not account for potential hormonal differences, placental effects, or hepatic Nrf2 expression changes a result of pregnancy. Thus, we propose the need to examine the placental transport of PCB126 parent compound in pregnant females with varying Nrf2 genotypes to assess the influence of placental oxidative stress and inflammation on offspring responses as a result of gestational PCB exposure. Ahmed et al. (28) reported that PCB126 gestational exposure disrupts placental tissues. Moreover, the current study would have benefited from an analysis of the glucose tolerance of Het and KO dams prior to and during pregnancy +/- PCB exposure. Further, one could argue that our experiments should have included WT comparisons or a Het/Het breeding scheme. We chose not to go this route because it would allow for us to look for maternal genotype differences, and the Het/Het breeding scheme would have produced many mice that were unnecessary. Our rationale for this study design is based on our preliminary analyses; we performed PCB exposure experiments in non-pregnant female WT and whole-body Het and KO mice and found that (1) PCB126 parent compound levels within the sera and livers of WT and Het animals were not significantly different, (2) AUC for blood glucose measurements did not differ between WT and Het animals (**Supplementary Figure 1**). It is important to note that our decision to eliminate WT animals from this study did not influence our ability to obtain sufficient data to answer our research question. Furthermore, the present study was designed to assess PCB-induced detriments in offspring using body composition analyses and glucose tolerance testing. This study could have benefited from employing insulin tolerance tests and serum measurements in addition to glucose stimulated insulin secretion assays to further validate the observed detrimental metabolic outcomes in offspring resulting from gestational PCB exposure. Lastly, future experiments should include a prolonged fasting period before glucose tolerance testing as well as utilize intraperitoneal administration of the glucose bolus to respectively tease out differences between groups and eliminate any potential effects of incretin hormones on insulin function and glucose homeostasis. In conclusion, this study demonstrates that *in utero* PCB exposure caused long-lasting alterations in offspring body weight, body composition, and glucose tolerance. This result reaffirms the importance of the timing of exposure and suggests that maternal interventions during the gestational period should be explored to lessen the negative effects of toxicant-induced health complications in offspring. Further, data from the current study show the influence of the maternal genotype on offspring response to early-life toxicant exposure. Although the developmental role of Nrf2 in PCB-toxicity is poorly understood, our work demonstrates that maternal allelic expression should not be ignored.

## Data Availability Statement

The raw data supporting the conclusions of this article will be made available by the authors, without undue reservation.

## Ethics Statement

The animal study was reviewed and approved by University of Kentucky Institutional Animal Care and Use Committee (IACUC).

## Author Contributions

BR, SN, and KP contributed to the conception and design of this study. BR, SN, OT, AA, FS, CC, SH, CH, NM, and KP contributed to data collection and/or interpretation of the data collected. BR SN, OT, AA, FS, CC, SH, CH, NM, HS, and KP contributed to the drafting of the manuscript and/or revising critically for important intellectual content. All authors have approved the manuscript and agree to be accountable for the content of the work.

## Funding

This study and core services were supported by US NIH grants (National Institute of General Medical Sciences, P20GM103527, and the National Institute of Environmental Health Sciences, P42ES007380 and P30ES026529). The Southern Regional Education Board Doctoral Scholars Program supported BR. CH was supported by the SURES (Summer Undergraduate Research in Environmental Health Sciences) program funded by NIEHS R25ES027684. NM was supported by Berea College Office of Internships & Career Development.

## Author Disclaimer

The content is solely the responsibility of the authors and does not necessarily represent the official views of the National Institutes of Health.

## Conflict of Interest

The authors declare that the research was conducted in the absence of any commercial or financial relationships that could be construed as a potential conflict of interest.

## Publisher’s Note

All claims expressed in this article are solely those of the authors and do not necessarily represent those of their affiliated organizations, or those of the publisher, the editors and the reviewers. Any product that may be evaluated in this article, or claim that may be made by its manufacturer, is not guaranteed or endorsed by the publisher.
