# Supplementary material for: Lack of Offspring Nrf2 Does Not Exacerbate the Detrimental Metabolic Outcomes Caused by In Utero PCB126 Exposure
Source: Front Endocrinol (Lausanne). 2021 Dec 16;12:777831. doi: 10.3389/fendo.2021.777831 (PMC8716916; doi:10.3389/fendo.2021.777831)
Supplement: Supplementary file 1 [file Presentation_1.pdf]

Supplementary Figure 1

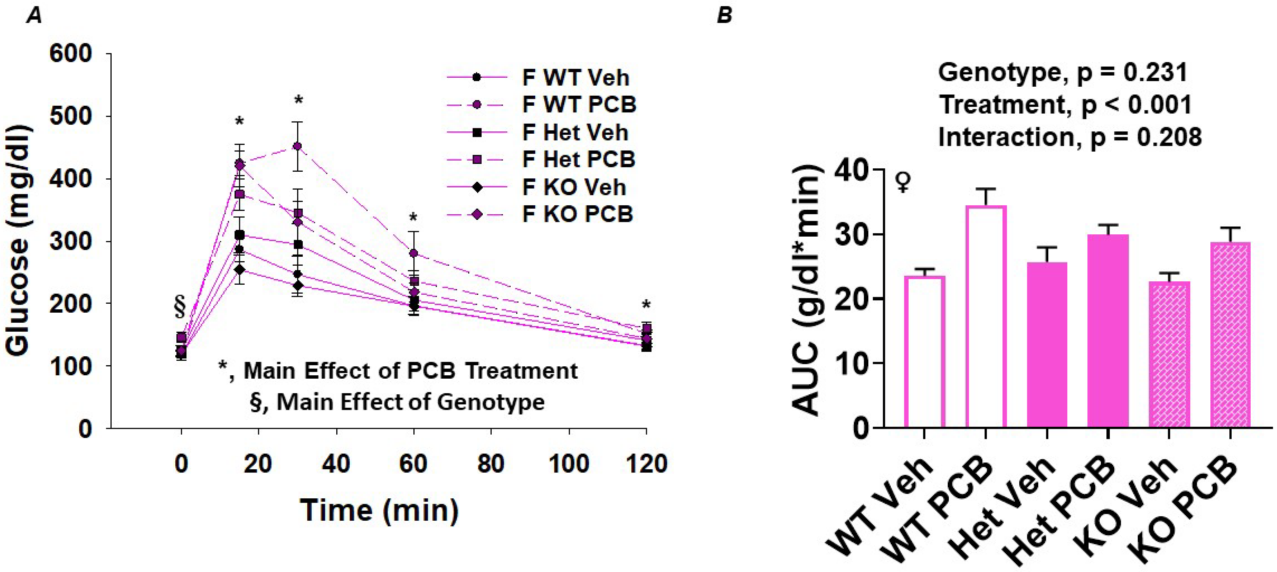

Supplementary Figure 1. Nrf2 genotype does not significantly alter glucose disposal in PCB-treated non-pregnant female mice. Non-pregnant female mice with varying whole-body Nrf2 genotypes were exposed to 1 µmol/kg of PCB126 at eight and ten weeks of age. Displayed are the data from the non-pregnant female mice 48 hours after their ten week PCB exposure. Glucose measurements **(A)** collected during the intraperitoneal glucose tolerance test were used to determine the area under the curve **(AUC; B)**. Values are indicative of the mean ± SEM (n = 5 per group). Significance was set at $\alpha = 0.05$. Two-factor repeated measures ANOVA and two-factor ANOVA were used respectively to detect differences in means of the glucose tolerance and AUC data. Significant main effects of PCB treatment (*) and genotype (§) are shown for Panel **(A)**.

Supplementary Figure 2

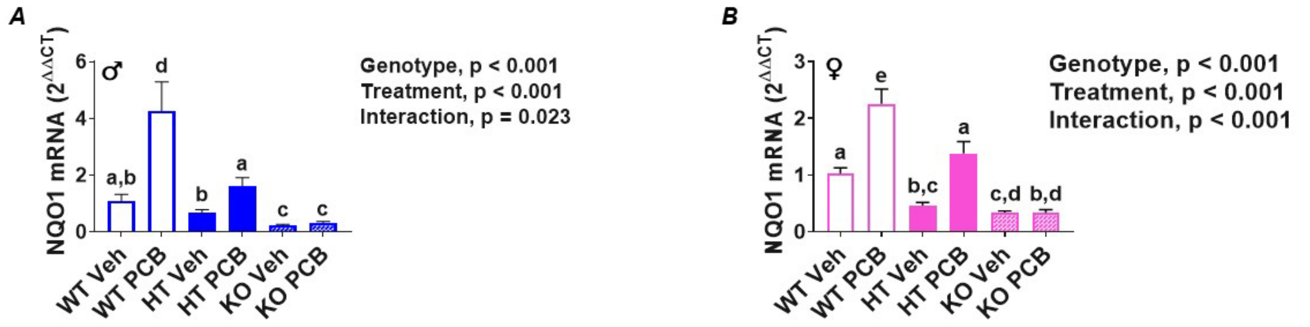

Supplementary Figure 2. **PCB126 exposure in Nrf2 wild-type, heterozygous, and knockout mice and hepatic expression of Nrf2 downstream target, NQO1.** At six weeks old, male **(A)** and female **(B)** mice of varying whole-body Nrf2 genotypes were exposed to 1 µmol/kg of PCB126 or vehicle. Twenty-four hours after exposure, animals were euthanized and the liver was harvested. RNA was isolated from the liver and mRNA levels of NQO1 were quantified. The mRNA levels of NQO1 were calculated using the ΔΔct method and is indicative of the fold differences of NQO1. Values displayed are the mean ± SEM (n = 5 per group). Significance was set at $\alpha = 0.05$. Two-factor ANOVA was used to detect differences in means. Upon detection of an interaction between genotype and treatment, one-factor ANOVA was employed to further detect differences between groups. Different letters indicate $p < 0.05$ between groups (i.e. bars labeled only with *a* are significantly different from bars labeled only with *b*, while neither is significantly different from a bar labeled with both an *a* and *b,* et cetera).
